# A Comparative Analysis of Traditional Self-Curing Resin Custom Tray and Thermoplastic Tray for Final Impressions in Edentulous Patients: A Pilot Study

**DOI:** 10.3390/jcm14248804

**Published:** 2025-12-12

**Authors:** Jong-Won Choi, Mi-Jung Yun, Il-Hwan Jang, Da-Hae Kim, You-jin Lee, Jung-Bo Huh

**Affiliations:** 1 Department of Prosthodontics, School of Dentistry, Pusan National University, Yangsan 50612, Republic of Korea; illuminus725@naver.com (J.-W.C.); p-venus79@hanmail.net (M.-J.Y.); jih4371@naver.com (I.-H.J.); edc205@naver.com (D.-H.K.); 2 Department of Prosthodontics, Dental Research Institute, Dental and Life Sciences Institute, Education and Research Team for Life Science on Dentistry, School of Dentistry, Pusan National University, Yangsan 50612, Republic of Korea

**Keywords:** 3-Dimensional (3D) analysis, thermoplastic, polycaprolactone, modeling compound, jaw, edentulous

## Abstract

**Background/Objectives:** This study aimed to compare the final impressions obtained using three different impression techniques in completely edentulous patients, focusing on the clinical applicability of the JB Tray^®^ for immediate custom tray fabrication with a polycaprolactone-based thermoplastic resin. **Methods:** Five edentulous patients were recruited for a preliminary clinical evaluation, and three impression techniques were tested: (1) JB Tray^®^ (JB), which allows simultaneous border molding and final impression taking; (2) the conventional modeling compound border-molded technique (MC, control); and (3) the one-step silicone border-molded technique (OS). All impressions were made by a single prosthodontist, and the internal surfaces were digitized using a 3D scanner for analysis of internal fit. Each arch was divided into reference regions for quantitative comparison. Statistical significance was set at *p* < 0.05. **Results:** Color map analysis (threshold of ±1.0 mm) revealed that the JB Tray^®^ group exhibited wider border extension in both the maxilla and mandible compared with the modeling compound and silicone groups, with a more pronounced effect in the mandible. However, no statistically significant differences in overall internal fit were observed among the three methods (*p* > 0.05). **Conclusions:** These preliminary results due to small sample size suggest that the JB Tray^®^ enables efficient impression taking in edentulous patients and can serve as a viable alternative to conventional custom tray techniques. Further research with a larger sample size is warranted to support widespread clinical application.

## 1. Introduction

The treatment of completely edentulous patients includes various prosthetic solutions, ranging from implant-supported restorations to conventional complete dentures [1,2,3,4,5,6,7,8,9]. However, due to systemic conditions and limited bone volume, extensive implant therapy is often precluded in the elderly population [8,9,10,11]. Consequently, conventional complete dentures remain the primary treatment modality, with substantial global demand [6,7,8,9,10,11].

The traditional method of fabricating complete dentures has been extensively validated over decades of clinical application [12,13,14,15]. This conventional workflow typically involves five major steps and necessitates a minimum of five patient visits [13,14,15]. The initial preliminary impression and the subsequent custom tray shape critically influence the final denture’s outcome [16,17,18]. Therefore, fabricating a well-adapted custom tray based on an accurate preliminary impression is paramount for successful denture construction. Obtaining an ideal preliminary impression—particularly of the challenging mandibular lingual area—remains clinically difficult, often requiring multiple adjustments to the custom tray and increasing chairside time [19,20,21]. With the advancement of digital technology, digital denture workflows are rapidly evolving [22,23,24,25]. Digital impressions allow for pressureless mucostatic recording and reproducible data storage. However, challenges persist, including soft tissue displacement during intraoral scanning and the difficulty of accurate border capture with optical impressions [26,27,28].

To overcome these limitations, the JB Tray^®^ (Just Border Tray, PNUADD, Busan, Korea) was developed to simplify clinical procedures and reduce the number of patient visits while preserving key principles of conventional denture fabrication. The JB Tray^®^ is a thermoplastic stock tray that becomes pliable when immersed in water over 70 °C, allowing intraoral adaptation and trimming to achieve a fit equivalent to that of a custom tray. The tray’s thick peripheral borders exhibit modeling compound-like properties, facilitating border molding. Once the impression is taken, the built-in vertical rods allow for immediate jaw relation records. In conjunction with the Pop-Bow^®^ (PNUADD, Busan, Korea), it also enables the transfer of occlusal plane and facial orientation information. As a result, the final denture can be delivered in as few as three clinical visits, even if a wax trial is necessary for occlusal verification. In clinical scenarios where clinicians attempt to trim stock trays or excessively modify ill-fitting custom trays, using the JB Tray^®^ to obtain a more accurate initial impression can offer clear advantages. However, to date, no studies have reported on the accuracy or specific characteristics of impressions obtained using JB Tray^®^ compared to conventional methods.

This study aimed to clinically compare final impressions obtained using traditional custom trays with silicone materials and modeling compound border molding, versus those obtained with the JB Tray^®^ without additional border molding procedure. Three-dimensional (3D) comparative analysis of the digitized impressions provides quantitative data and visualizes the internal fit (intaglio surface accuracy) as well as the length and thickness of the border extension. This quantitative and visualized feedback will guide clinicians in the appropriate utilization and adaptation of the JB Tray^®^ in clinical practice. The null hypothesis was that “there is no significant difference between edentulous impressions obtained using the traditional custom tray and those obtained with the JB Tray^®^”.

## 2. Materials and Methods

### 2.1. Description of the Medical Device and Clinical Procedure (JB Tray^®^)

**Figure 1:**
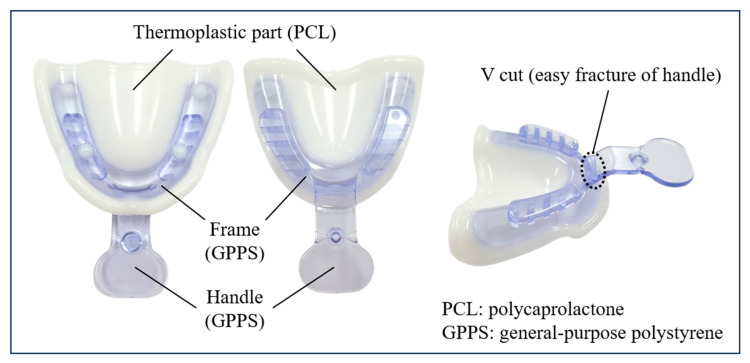
Description of the Upper (maxillary) JB Tray^®^.

**Figure 2:**
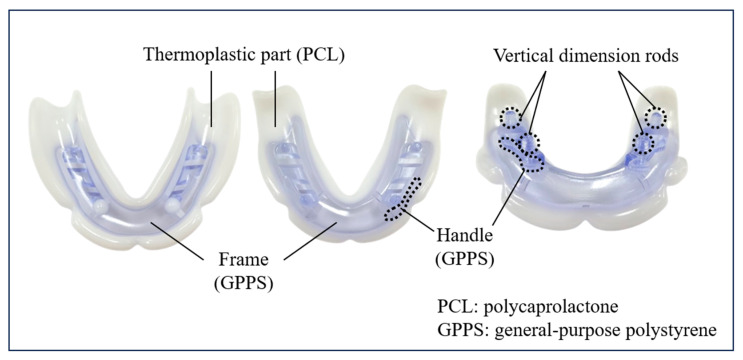
Description of the Lower (mandibular) JB Tray^®^.

The JB Tray^®^ (Just Border Tray; PNUADD, Busan, Korea) is a thermoplastic stock tray designed for same-day final impressions and jaw relation recording. The maxillary tray resembles a conventional edentulous tray with a handle (Figure 1), while the mandibular tray lacks an anterior handle to facilitate closed-mouth techniques and features four vertical rods for establishing vertical dimension (Figure 2).

Each tray consists of a general-purpose polystyrene (GPPS) base extending along the alveolar ridge, integrated with a thermoplastic polycaprolactone (PCL) border. The white PCL border softens and becomes transparent when immersed in water above 70 °C, allowing for intraoral adaptation. Upon cooling, it returns to a rigid white state, exhibiting mechanical properties similar to modeling compound. The tray can be reheated and reshaped multiple times as needed.

**Figure 3:**
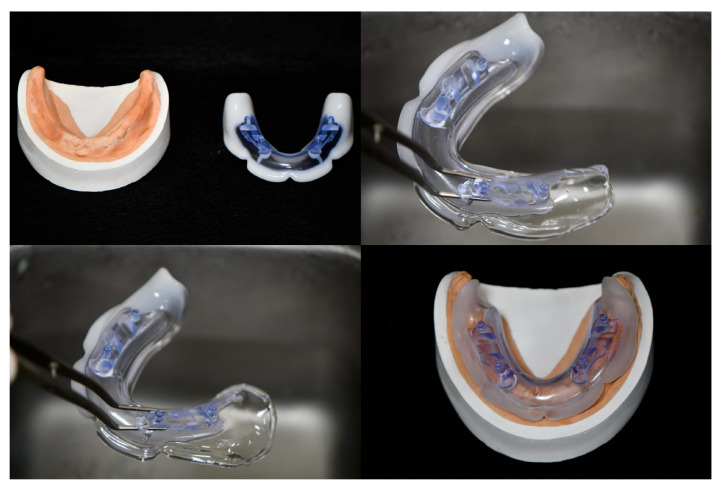
Illustration of customization process of a JB Tray^®^ for an edentulous mandibular model. (**Top Left**): The edentulous mandibular model and the initial JB Tray^®^ are shown. (**Top Right**): The tray’s color change after immersion in hot water (approximately 70 °C) confirms its easy deformability via light manual manipulation. (**Bottom Left**): The JB Tray^®^ after light stretching, showing the distal extension of the posterior region. (**Bottom Right**): The JB Tray^®^ applied to the edentulous mandibular model, demonstrating the proper adaptation of both the border (periphery) and the intaglio surface to the ridge.

Polycaprolactone (PCL) is a biodegradable synthetic polyester known for its high biocompatibility. Due to these properties, it has been used in various medical applications, including surgical sutures and fillers, and in dentistry for appliances such as night guards and root canal filling materials. The oral conditions of edentulous patients are highly individualized, varying significantly in factors such as bone volume (dependent on the timing of residual tooth extraction) and the type of opposing dentition. Consequently, the traditional method of manufacturing an impression tray involved taking an impression and then fabricating the tray extra-orally. Even when using a stock tray, customizing it intra-orally, for instance by using putty, has the disadvantage of being time-consuming. PCL offers several advantages: it has a low melting point, exhibits stable volumetric stability upon hardening, and when combined with appropriate materials, allows for a long working time. These characteristics make it possible to quickly and easily convert it into a custom tray within the patient’s mouth. (Figure 3).

After achieving a proper fit, silicone adhesive(3M VPS tray adhesive; 3M, St. Paul, MN, USA) is applied, and polyvinyl siloxane (Exadenture; GC, Tokyo, Japan) material is used for final impressions. Once the maxillary impression is completed, the handle is removed to enable mandibular closed-mouth impressions. For the mandibular tray, border molding is performed intraorally using functional tongue movements; if difficult, pre-shaping is performed extra-orally for 30 s before reinsertion.

Following border molding, vertical dimension is adjusted using the tray’s vertical rods to ensure at least two occlusal contact points. Once a stable occlusion is confirmed, mandibular impressions are made with PVS material, and centric relation and vertical dimension are recorded. Facial reference and occlusal plane orientation are transferred using the Pop-Bow^®^ (PNUADD, Busan, Korea), which is attached to the tray with silicone material. This protocol supports same-day denture fabrication via 3D printing for interim prostheses, while final dentures can be delivered in two to three visits depending on the need for wax trial verification.

### 2.2. Clinical Study Design

This study was conducted on five edentulous volunteers (two men and three women; mean age, 76.4 years) at Pusan National University Dental Hospital. Power analysis indicated a moderate-to-large effect size (ε2 = 0.204). A post hoc power analysis using G*Power(ver. 3.1.9.2) yielded a result in a statistical power of approximately 0.78, suggesting that the sample size of five patients was sufficient to ensure adequate statistical power for this study. But the results should be interpreted cautiously as preliminary findings, not definitive clinical claims. All clinical procedures followed ethical guidelines approved by the Institutional Review Board and Medical Device Clinical Trial Committee (IRB No.: PNUDH IRB 2024-11-013). Informed consent was obtained from all participants after a thorough explanation of the study’s purpose, procedures, and compensation. Patient demographic data, including age and gender, are detailed in Table 1.

Inclusion criteria were as follows:

Completely edentulous patients aged 65 years or older requiring new dentures;

A minimum of 6 months of healing after tooth extraction with stable alveolar ridges;

No evident gingival inflammation, swelling, or bleeding;

No impairment in masticatory function;

Ability to maintain proper oral hygiene.

All impressions were made in an open-mouth position to eliminate potential variation from different impression techniques. Each patient underwent three impression techniques for both the maxilla and mandible, performed by a single prosthodontist (over 10 years of prosthodontic practice) to minimize inter-operator variability.

**Figure 4:**
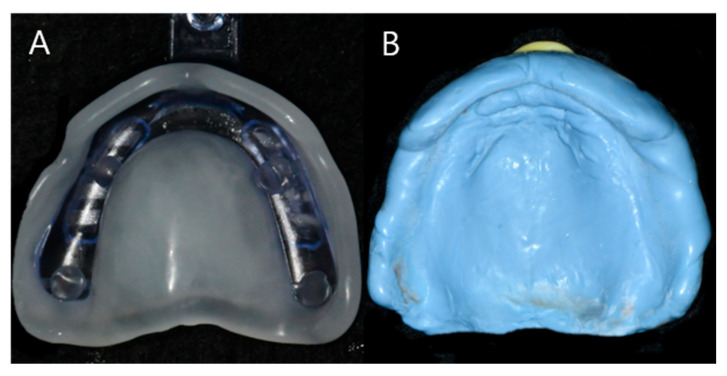
Maxillary impression of Group JB, Internal fit and border adjustment with JB Tray^®^ (**A**), Final impression of Gorup JB (**B**).

**Figure 5:**
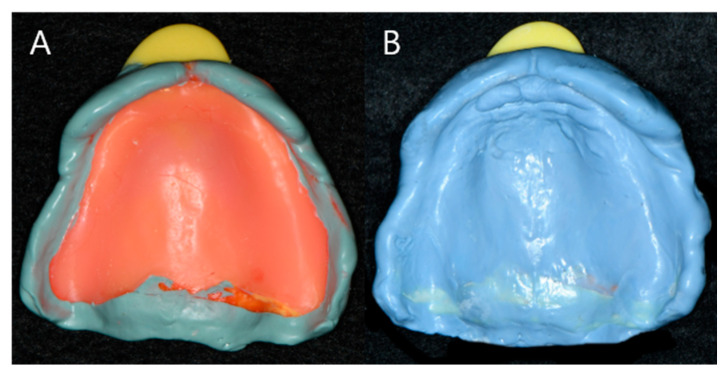
Maxillary impression of group OS, Border adjustment with silicone impression material (**A**), Final impression of group OS (**B**).

**Figure 6:**
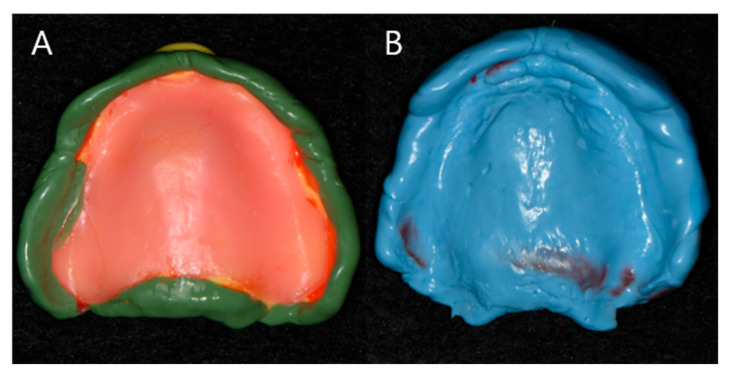
Maxillary impression of Group MC, Border adjustment with modeling compound material (**A**), Final impression of group MC (**B**).

(1) JB Tray^®^ Group (JB): Final impressions were made using JB Tray^®^ with thermoplastic border molding and no additional materials (Figure 4)
(2) One-Step Silicone Group (OS): Border molding was performed on self-curing resin custom trays using silicone-based material (Exahiflex; GC, Tokyo, Japan) (Figure 5)
(3) Modeling Compound Group (MC): Border molding was performed on self-curing resin custom trays using modeling compound (Peri Compound, GC, Tokyo, Japan) (Figure 6).

The same silicone impression material (Exadenture; GC, Tokyo, Japan) was used for all groups.

### 2.3. Clinical Procedure of Edentulous Impression

**Figure 7:**
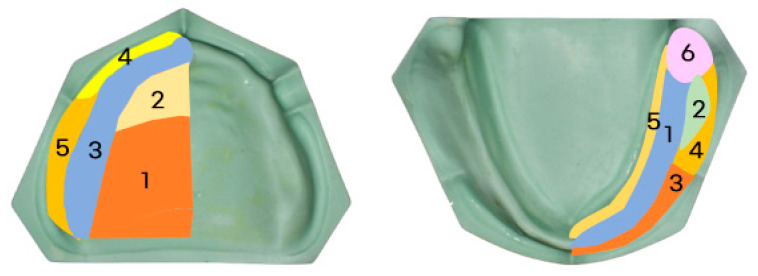
Segmented Reference zones in Maxilla and Mandible; (Maxilla) 1. Posterior palate, 2. Anterior palate, 3. Alveolar ridge, 4. Labial flange, 5. Buccal flange, (Mandible) 1. Alveolar ridge, 2. Buccal shelf, 3. anterior buccal flange, 4. Posterior buccal flange, 5. Lingual flange, 6. Retromolar pad area.

**Figure 8:**
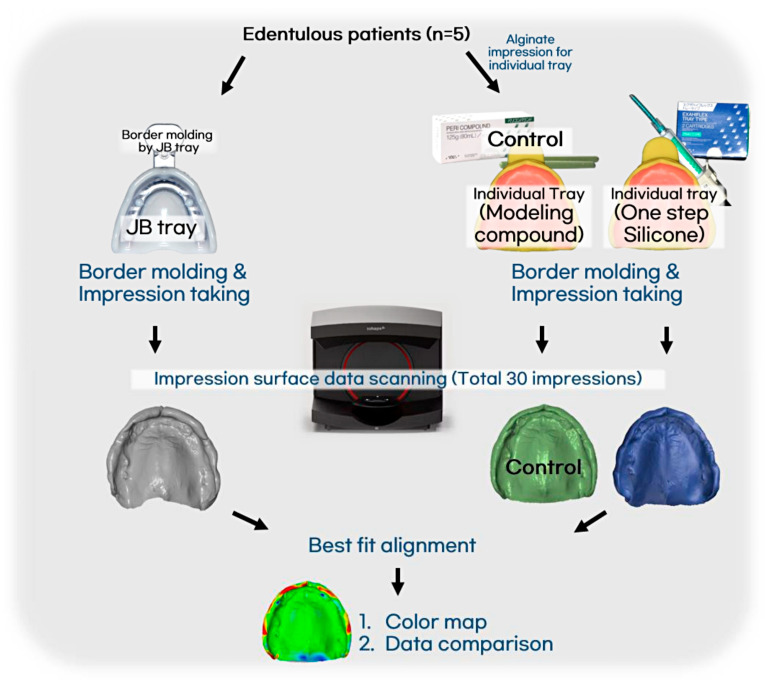
Protocol of this experiment followed by five patients.

Each patient underwent all three methods, resulting in a total of 15 pairs of maxillary and mandibular final impressions. On the first visit, JB group were performed using 70 °C water baths to soften the tray borders. Most of the internal surface and border adaptation of the JB Tray^®^ was performed after a single heating cycle; however, in the mandible, re-adaptation was performed once with partially reheating of the border, when deemed necessary. Followed by trimming border to 1 mm uniform space and final impression with silicone (Exadenture; GC, Tokyo, Japan). Additionally, alginate preliminary impressions were taken to create diagnostic casts. Based on these casts, two custom trays were fabricated per patient, with selective relief applied (posterior palatal dam for maxilla, buccal shelf for mandible) and border outlines defined. At the second visit, border molding was performed using silicone-based border molding material (Exahiflex; GC, Tokyo, Japan) and modeling compound(Peri Compound, GC, Tokyo, Japan), respectively, following conventional techniques. All procedures were carried out consistently by a single operator. All trays were made 2 mm shorter than the expected final denture border. The fit was verified with Fit Checker (GC, Tokyo, Japan) and uniformly reduced by 1 mm at the margin after border molding. Final impressions were taken with silicone material (Exadenture; GC, Tokyo, Japan) for both arches. For each patient, three different types of impressions were obtained and scanned individually using a desktop scanner (E3; 3Shape, Copenhagen, Denmark), and STL files were generated. Best-fit alignment and color mapping for visual evaluation of internal adaptation were conducted using GOM Inspect (version 2018; ZEISS, Braunschweig, Germany). Quantitative evaluation of internal fit accuracy was performed using Geomagic Control X (version 2017.0.3; Oqton, Ghent, Belgium). Each arch was divided into five (maxilla) and six (mandible) reference zones to assess displacement differences across regions as shown in Figure 7. A schematic of the entire process is summarized in Figure 8.

### 2.4. Statistical Analysis

Statistical analysis was conducted using SPSS software (version 27.0, SPSS Inc., Chicago, IL, USA). The non-parametric Kruskal–Wallis test was applied to compare the impression methods across the five patients, with Mann–Whitney U tests used for post hoc analysis. Statistical significance was set at *p* < 0.05.

## 3. Results

### 3.1. Color Map Comparison

**Figure 9:**
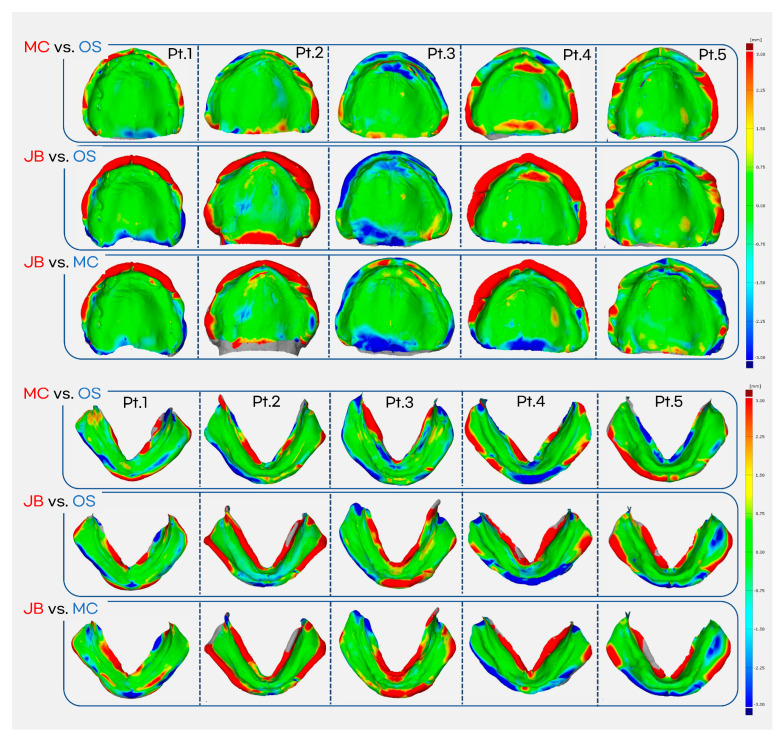
Color map generated using GOM Inspect software (±1 mm scale bar applied). MC vs. OS comparison is superimposed on the OS impression, while all other comparisons are superimposed on the JB impression. Red indicates overextension or increased internal pressure (positive deviation). Green represents minimal differences. Blue indicates shorter marginal areas or reduced internal pressure (negative deviation).

Color maps comparing the impression materials are presented in Figure 9, generated using GOM Inspect software. Scan artifacts was removed manually, and best-fit alignment on global area was performed using the software’s automatic registration function, and discrepancies between impressions were visualized by color differences. To enhance the clinical interpretation of these small deviations, we defined an explicit clinical relevance threshold of ±1.0 mm for the 3D superimposition, and the differences observed were largely below this threshold. In the maxilla, the MC group showed a slightly more extended impression margin compared to the OS group, while the JB group demonstrated further extension compared to the MC group. In the mandible, the MC group exhibited marginal extension at some lingual areas compared to the OS group, and the JB group showed additional extension at both buccal and lingual margins compared to the MC group. No significant differences were observed in the intaglio surface among the groups.

### 3.2. Comparison of Deviation and RMS Value

Maxillary impression comparison

**OS vs. MC** (Table 2) The superimposition of OS onto the control MC for the maxilla showed that OS had a more extended margin and was more depressed compared to MC, with an overall average difference of 0.09 ± 0.15 mm. (Positive values indicate more extended margin/compression by MC, negative by OS.) The largest difference (0.45 ± 0.34 mm) and the highest RMS value (0.87 ± 0.24 mm) were both found in the buccal flange (region 5), indicating greater peripheral extension for MC in this area. Overall RMS value was 0.45 ± 0.09 mm. Differences in other regions (posterior palatal, region 1; anterior palatal, region 2; alveolar ridge, region 3; and labial flange, region 4) were minimal (ranging from −0.02 to 0.01 mm).

**JB vs. MC** (Table 3) The superimposition of JB onto the control MC showed that JB had greater border extension and was more depressed overall compared to MC, with an average difference of −0.04 ± 0.18 mm. (Negative values indicate more extended margin/compression by JB.) The largest average difference (−0.42 ± 0.38 mm) and the largest RMS value (1.13 ± 0.59 mm) were observed at the labial flange (region 4). Overall RMS value was 0.59 ± 0.21 mm. Other regional differences were minimal (ranging from 0.003 to 0.16 mm).

Mandibular impression comparison

**OS vs. MC** (Table 4) For the mandible, MC showed more notable border extension but tended to exert less pressure on the intaglio surface compared to OS, with an overall difference of 0.05 ± 0.12 mm. (Positive values indicate more extended margin/compression by MC, negative by OS.) The lingual flange (region 5) showed the largest average difference (0.43 ± 0.43 mm), suggesting a more extended margin for MC in this region. This region also had the largest RMS value (1.25 ± 0.20 mm). The overall RMS value was 0.78 ± 0.06 mm.

**JB vs. MC** (Table 5) The superimposition of JB onto the control MC for the mandible showed that JB tended to have an extended margin at the lingual and labial flanges compared to MC, resulting in an overall difference of −0.14 ± 0.29 mm. (Negative values indicate more extended margin/compression by JB.) The largest average difference (−0.61 ± 0.21 mm) and the largest RMS value (1.55 ± 0.21 mm) were found at the lingual flange (region 5), indicating greater extension by the JB Tray^®^. The overall RMS value was 0.95 ± 0.15 mm.

In conclusion, in a comparison of the intaglio surfaces, no significant differences were observed among the three impression techniques for both the maxilla and mandible. Regarding the comparison of border areas, the difference between impression methods was greater in the mandible than in the maxilla. MC showed greater extension on margin than OS, and JB showed more extension on margin than MC at the maxillary posterior buccal margin and the mandibular lingual margin. No statistically significant differences were found between three impression methods (*p* > 0.05).

## 4. Discussion

In this study, the null hypothesis, suggesting differences in edentulous impression taking among the JB, OS, and MC was not rejected. While the MC group showed a slight tendency towards extended borders compared to the OS group, and the JB group exhibited slightly greater border extension than the MC group. However, the overall average differences in both comparisons were minimal and not statistically significant, a finding that should be interpreted cautiously given the limited sample size (*n* = 5).

Superimposition analysis revealed that differences among the impression techniques were more prominent in the mandible than in the maxilla. In the maxilla, MC showed slightly more extended borders than OS, particularly in the buccal flange, while JB exhibited a marginally greater extension than MC, especially in the labial flange. In the mandible, MC showed greater border extension than OS, especially along the lingual flange, and exerted less internal pressure overall. Similarly, JB showed even greater extension than MC at both the lingual and labial flanges. The largest RMS differences across all comparisons were consistently observed in these regions, indicating higher variability in border adaptation for mandibular impressions. These findings indicate that mandibular borders -especially the lingual flange-are more susceptible to variations in impression technique and material. Although JB and MC tended to produce more extended borders than OS, especially in critical areas related to denture retention, the differences were not statistically significant. Nevertheless, the clinical relevance of lingual extension for mandibular denture stability should not be underestimated, and careful control of border molding remains essential [13]. During impression, borders of JB Tray^®^ group were uniformly reduced by 1 mm after border molding. Although this is not a standard clinical step, it was performed to minimize variability caused by manual adjustment during border trimming and to assess the border molding ability of JB Tray^®^ material. In the actual clinical practice, incorporating an additional verification step—such as using a fit checker—could further enhance the precision and reliability of border adaptation. When comparing the differences in border molding materials among traditional impression techniques, in maxillary impressions, the use of silicone border molding material did not result in significant differences compared to modeling compound. However, in mandibular impressions—particularly in forming the lingual border—some cases in the silicone group showed shortened or thinned borders. In several instances, the border material tore during the 1 mm reduction process, making evaluation difficult. In mandible, considering that adequate extension in sublingual space and 4–5 mm below the mylohyoid ridge is crucial for denture stability [13,14,15,29], modeling compound appears more favorable than silicone for lingual border formation. On the other hand, overextension or overly steep inclines in the lingual flange can interfere with mylohyoid muscle movement during swallowing, potentially causing discomfort [29]. Therefore, even with the JB Tray^®^, careful evaluation and adjustment of the internal surface following border molding are essential to prevent overextension, which can cause clinical issues like pain and compromised denture retention.

Clinical Workflow Implications of the JB Tray^®^

While the application of digital technology in edentulous impressions has shown superior results regarding the intaglio surface, there are significant difficulties in establishing the borders [28,30]. Consequently, it is challenging to directly utilize digital impressions for the fabrication of definitive dentures, leading to a common practice of replicating existing dentures for impression purposes. In this context, the JB Tray^®^ offers an integrated approach because it allows for the integration of digital workflows, aimed at reducing patient visits and enhancing operator convenience, without completely bypassing the traditional impression process.

The JB Tray^®^ functions similarly to a custom tray in shaping the internal surface of the impression. Like modeling compound, its thermoplastic border can be reheated in warm water to provide sufficient working time and can be easily molded by hand or functional muscle movements. Furthermore, compared to conventional open-mouth impressions, the JB Tray^®^ captures a broader area—including the polished surface—which could potentially eliminate the need for dental technicians to arbitrarily determine the contour and thickness of the denture flange. Clinically, the JB Tray^®^ is suggested to have several advantages over conventional methods. Maxillary impression can be taken using the open-mouth technique with the JB Tray^®^. The handle can then be detached to allow for a closed-mouth mandibular impression. The vertical pin can be adjusted to establish vertical dimension, and the intermaxillary relationship can be recorded immediately using Alu-wax or silicone bite material. This integrated workflow simplifies the conventional complete denture process, which traditionally requires at least five appointments, potentially benefiting both clinicians and patients. However, compared to the traditional approach using custom trays and occlusal rims, the JB Tray^®^ system requires a deeper understanding of denture principles and may require a learning period before becoming fully proficient in its issue.

Methodological Considerations and Limitations

The results of this study revealed no statistically significant differences between impressions taken with the JB Tray^®^ and those taken with conventional custom trays using the open-mouth technique. Therefore, the JB Tray^®^ appears to be a clinically viable option for edentulous impressions, providing practical benefits for both clinicians and patients. Notably, it eliminates the need for an additional appointment for custom tray fabrication and model delivery to the laboratory, potentially reducing the total number of clinical visits.

However, this exploratory study had limitations. There was no absolute reference model for comparing the internal surfaces of the impressions. Resin markers occasionally detached during the impression process, requiring optimal manual alignment during superimposition. Although the manual trimming of scan artifacts on individual impressions was performed by a single calibrated clinician, this operator-dependent step may nonetheless introduce variability and potentially influence the reproducibility of the data. Closed-mouth impressions, though more challenging in terms of pressure control and dependent on patient cooperation, have been reported to improve patient satisfaction and retention in mandibular cases [31]. Given that the JB Tray^®^ allows for closed-mouth mandibular impressions, the strict use of the open-mouth technique in this study was solely for standardization across all three groups; future comparative studies with denture systems such as BPS should utilize the closed-mouth technique for mandibular impressions to better reflect the JB Tray’s clinical utility. Also, it was difficult to quantify the exact pressure exerted on the tissues or the degree of border extension. Operator technique likely played a significant role in impression outcomes. Additionally, the study was conducted on only five patients, and each underwent six impressions, which was burdensome for both patients and the operator. Furthermore, regarding the sequence of impressions, the JB Tray^®^ impression was necessarily performed first to avoid an additional patient visit, as it integrates the preliminary and final impression steps. The custom tray impressions, which require a separate preliminary impression appointment, were subsequently performed in random order. However, this fixed sequence for the initial JB Tray^®^ impression may have introduced a systematic bias or order effect which should be acknowledged as a methodological limitation. It is important to note that the scope of this study was limited strictly to comparing the accuracy of the impressions. Evaluating factors such as denture retention, stability, and patient comfort requires fully fabricated dentures and comprehensive patient feedback after adequate use, which involves too many confounding variables outside of the impression technique, including the stability of the recorded jaw relationship, the arrangement of artificial teeth, and errors during denture polymerization. Subsequent research is currently underway to address these clinical outcomes. Future research must include a larger, more heterogeneous sample size, establish correlation with objective clinical outcome measures (e.g., retention tests, patient comfort), and evaluate the accuracy of intermaxillary record registration using the JB Tray^®^, as this is equally critical to successful complete denture fabrication.

## 5. Conclusions

Although no statistically significant differences were found among the three impression techniques, both the maxillary labial and mandibular lingual borders tended to exhibit greater extension in the MC group than in the OS group, and even further in the JB Tray^®^ group. These tendencies indicate that, when using the JB Tray^®^ for final impressions, careful verification of border adaptation and internal surface accuracy is advisable to prevent potential overextension.

## Figures and Tables

**Table 1:** Patient Demographics.

Characteristic	Value
Sample Size (*n*)	5
Gender (Male:Female)	2:3
Mean Age (Years)	76.4 y
Age Range (Years)	[68 y–82 y]
Inclusion Criteria Compliance	All participants

**Table 2:** Comparison of marginal discrepancy (mm) between MC and OS in the Maxilla.

Area	Mean ± SD	Positive Deviation from Control (mm)		Negaitive Deviation from Control (mm)		RMS ± SD
		Maximum	Mean Positive	Minimum	Mean Negative	
Area 1	−0.02 ± 0.04	0.93	0.15	−1.18	−0.16	0.25 ± 0.09
Area 2	0.01 ± 0.24	0.79	0.17	−0.74	−0.17	0.29 ± 0.23
Area 3	0.01 ± 0.03	0.93	0.15	−0.89	−0.14	0.21 ± 0.10
Area 4	−0.03 ± 0.37	1.76	0.46	−1.98	−0.44	0.65 ± 0.08
Area 5	0.46 ± 0.34	2.32	0.74	−1.58	−0.35	0.87 ± 0.24
overall	0.09 ± 0.16	1.35	0.34	−1.27	−0.25	0.45 ± 0.09

**Table 3:** Comparison of marginal discrepancy (mm) between MC and JB in the Maxilla.

Area	Mean ± SD	Positive Deviation from Control (mm)		Negative Deviation from Control (mm)		RMS ± SD
		Maximum	Mean Positive	Minimum	Mean Negative	
Area 1	0.11 ± 0.10	1.70	0.30	−1.44	−0.14	0.32 ± 0.19
Area 2	−0.07 ± 0.06	0.12	0.10	−0.33	−0.18	0.20 ± 0.07
Area 3	0.00 ± 0.05	1.50	0.19	−1.49	−0.20	0.31 ± 0.07
Area 4	−0.42 ± 0.80	2.10	0.36	−3.05	−0.91	1.14 ± 0.60
Area 5	0.16 ± 0.38	2.63	0.69	−2.68	−0.63	0.99 ± 0.32
overall	−0.04 ± 0.18	1.61	0.33	−1.80	−0.41	0.59 ± 0.21

**Table 4:** Comparison of marginal discrepancy (mm) between MC and OS in the Mandible.

Area	Mean ± SD	Positive Deviation from Control (mm)		Negative Deviation from Control (mm)		RMS ± SD
		Maximum	Mean Positive	Minimum	Mean Negative	
Area 1	−0.03 ± 0.03	0.89	0.16	−1.21	−0.20	0.27 ± 0.09
Area 2	−0.04 ± 0.07	0.72	0.15	−1.09	−0.21	0.25 ± 0.07
Area 3	0.06 ± 0.06	3.39	0.86	−2.42	−0.69	1.12 ± 0.19
Area 4	−0.29 ± 0.50	1.57	0.59	−2.69	−0.79	0.93 ± 0.14
Area 5	0.43 ± 0.44	4.03	1.13	−3.37	−0.65	1.26 ± 0.20
Area 6	−0.02 ± 0.26	3.02	0.63	−2.72	−0.66	0.89 ± 0.22
overall	0.05 ± 0.12	2.30	0.58	−2.14	−0.49	0.78 ± 0.06

**Table 5:** Comparison of marginal discrepancy (mm) between MC and JB in the Mandible.

Area	Mean ± SD	Positive Deviation from Control (mm)		Negative Deviation from Control (mm)		RMS ± SD
		Maximum	Mean Positive	Minimum	Mean Negative	
Area 1	−0.04 ± 0.08	0.84	0.13	−1.2	−0.18	0.25 ± 0.10
Area 2	−0.10 ± 0.17	0.65	0.17	−1.39	−0.28	0.39 ± 0.20
Area 3	−0.46 ± 1.03	2.51	0.59	−3.97	−1.03	1.32 ± 0.58
Area 4	0.24 ± 0.89	1.91	0.70	−1.96	−0.5	1.04 ± 0.45
Area 5	−0.61 ± 0.21	4.51	1.12	−4.76	−1.21	1.55 ± 0.21
Area 6	0.14 ± 0.41	3.57	0.83	−3.03	−0.43	1.13 ± 0.17
overall	−0.14 ± 0.29	2.33	0.59	−2.72	−0.61	0.95 ± 0.15

## Data Availability

All data have been presented in the manuscript.
